# Metabolic effect of drought stress on the leaves of young oil palm (*Elaeis guineensis*) plants using UHPLC–MS and multivariate analysis

**DOI:** 10.1038/s41598-021-97835-x

**Published:** 2021-09-14

**Authors:** Jorge Candido Rodrigues Neto, Letícia Rios Vieira, José Antônio de Aquino Ribeiro, Carlos Antônio Ferreira de Sousa, Manoel Teixeira Souza Júnior, Patrícia Verardi Abdelnur

**Affiliations:** 1 Institute of Chemistry, Federal University of Goiás, Campus Samambaia, Goiânia, GO 74690‐900 Brazil; 2 Graduate Program of Plant Biotechnology, Federal University of Lavras, CP 3037, Lavras, MG 37200-000 Brazil; 3 Brazilian Agricultural Research Corporation, Embrapa Agroenergy, Brasília, DF 70770‐901 Brazil; 4 Brazilian Agricultural Research Corporation, Embrapa Mid-North, Teresina, PI 64008-780 Brazil

**Keywords:** Chemistry, Analytical chemistry, Plant sciences, Plant biotechnology

## Abstract

The expansion of the oil palm in marginal areas can face challenges, such as water deficit, leading to an impact on palm oil production. A better understanding of the biological consequences of abiotic stresses on this crop can result from joint metabolic profiling and multivariate analysis. Metabolic profiling of leaves was performed from control and stressed plants (7 and 14 days of stress). Samples were extracted and analyzed on a UHPLC-ESI-Q-TOF-HRMS system. Acquired data were processed using XCMS Online and MetaboAnalyst for multivariate and pathway activity analysis. Metabolism was affected by drought stress through clear segregation between control and stressed groups. More importantly, metabolism changed through time, gradually from 7 to 14 days. The pathways most affected by drought stress were: starch and sucrose metabolism, glyoxylate and dicarboxylate metabolism, alanine, aspartate and glutamate metabolism, arginine and proline metabolism, and glycine, serine and threonine metabolism. The analysis of the metabolic profile were efficient to correlate and differentiate groups of oil palm plants submitted to different levels of drought stress. Putative compounds and their affected pathways can be used in future multiomics analysis.

## Introduction

Palm oil, derived from the African Oil Palm (*Elaeis guineensis* Jacq.), is the most consumed edible oil in the World, with a global production of 83.96 million metric tons—palm oil and palm kernel oil—in 2020/2021^1^. This crop is highly dependent on water availability; therefore, drought stress could represent a high risk on the production yield. In the next few decades, the population growth and subsequently vegetable oil demands could lead to the unforeseen expansion of palm tree crops. However, limiting factors such as abiotic stresses are present in most potential farmable areas^12^.

Water withhold directly affects the plant metabolism, given that defense mechanisms are promptly activated to reduce the implications of the stress. Usually, abiotic stress responses are related to crop growth, cell development, CO_2_ fixation, photosynthesis capability, etc.^2^. Drought stress also induces the production and activation of compounds that modulate certain metabolites and pathways, e.g., cell homeostasis^3^.

Metabolomics is a powerful tool to study applied stresses in plants due to the high capacity of compounds detection, identification, and pathway correlation through different methods^4–6^. This technique is described as a “snapshot” of the studied organism, illustrating which compounds are present and their concentrations. The challenges faced on metabolomics analysis relies mainly on the complex biological matrices, which require different extraction and analytical techniques in order to detect, identify and/or quantify the highest possible number of metabolites^5^.

The plant response to an environmental interaction such as drought stress is an enormous array of chemically altered metabolites. Metabolomics fits the abiotic stress study demand because metabolites are the most direct representation of the plant phenotype, since they are signatures of the biological and chemical activity^3^. Therefore, in order to lead stress tolerance studies in plants, there is a surging interest to observe the metabolite level changes after the abiotic stress^4,6^.

Although many analytical techniques can be successfully employed in a metabolomics study, chemical separation and detection mainly resolves around nuclear magnetic resonance and mass spectrometry. Liquid chromatography is, in most cases, the choice adequate for polar phytochemical compound separation, even from complex matrices. Mass spectrometry offers a coupled technique (LC–MS) to detect and identify metabolites using high resolution and selectivity^7^. This tandem method is applied successfully to analyze a vast array of metabolites in plants, from different chemical classes—flavonoids, alkaloids, glucosinolates, organic acids, and others^4,5,8–10^.

Discovering data patterns are a difficult task when done manually; therefore, a statistical treatment is necessary. The capability to organize and visualize high amounts of data comes from supervised classification methods, such as partial least square discriminant analysis (PLS-DA), which provides group separation based on their mass profile. Supervised methods bring the ability to reduce spatial components with no information loss, therefore metabolites detected and inserted in this model can be grouped through regression, which amplifies the discrimination between samples and visually defines groups with different treatments. Metabolic pathways can be further related to the grouped samples with the use of algorithms such as mummichog^11^ to improve the biological meaning of the experiment.

Young oil palm leaves were submitted to metabolic fingerprinting analysis using ultra-high-performance liquid chromatography–electrospray ionization–mass spectrometry (UHPLC–ESI–MS) for detection of polar compounds. Data analysis from MS spectra was performed through statistical visualization using PLS-DA, heatmap, and pathway activity analysis.

Therefore, the aim of this study is to present a high-throughput untargeted method to identify drought-related metabolic pathways to improve the knowledge about oil palm response, which will be useful in further multiomics studies.

## Results and discussion

### Biochemical, morphophysiological responses and differential expression analysis: contextualization and data correlation

The current study derives from previous research activities on the characterization of the morphophysiological responses and analysis of differentially expressed genes of oil palm to drought stress^12^. Some results of these activities will be used in the future to corroborate and compare with the biochemistry of oil palm drought stress. Important parameters showed that non-irrigated plants were physiologically stressed and such stress could be responsible for metabolic changes. We have collected information regarding evapotranspiration and soil water potential, leaf gas exchange [net CO_2_ assimilation rate (A), transpiration rate (E), stomatal conductance to water vapor (gs), and intercellular CO_2_ concentration (Ci)], chlorophyll fluorescence [Fm, Fo, Y(II), Fv/Fm, Y(NPQ), and Y(NO)], pigment content, leaf relative water content and leaf temperature (including thermographic images). This data is not shown at this moment as it has been integrated to mRNA and miRNA transcriptome data for future studies.

The drought-stressed plants suffered a gradual reduction in water content from the substrate, resulting in a fall of the soil water potential, evapotranspiration rate, and fresh biomass. The net CO_2_ assimilation, stomatal conductance, and transpiration rates suffered a statistical reduction. The fall in net CO_2_ assimilation and stomatal conductance rates, which led to a reduction or inhibition of the enzymatic activity, is the cause of this decrease in photosynthetic activity^13,14^. Therefore, the unbalance caused by the low water availability can directly affect the cellular metabolism given the excess or lack of essential metabolites needed for the plants' biochemical reactions.

In a state of water deprivation, plants usually suffer function rates and photosynthetic efficiency alteration^15,16^. *E. guineensis* samples presented a linear decrease in chlorophyll concentration and factors related to chlorophyll fluorescence only after the 11th day of drought stress.

These data led us to infer that some analyses are better for stress detection, depending on the level of sensibility. After irrigation interruption, many cellular metabolism alterations can be detected by high throughput phenotyping methods, depending on intensity, time of exposure, developmental stage, and species analyzed^17,18^. In this study, the metabolomics approach fits due to the drought sensitivity presented just a few days after the start of the water deprivation.

### Metabolic fingerprinting analysis

Metabolic fingerprinting is widely known as a powerful untargeted approach that correlates chromatogram profiles and the compound information within the MS peaks. The drought stress was studied by comparison of the metabolic profile in plants of three groups: control (irrigated) and stressed samples (7 and 14 days of water deprivation).

**Figure 1:**
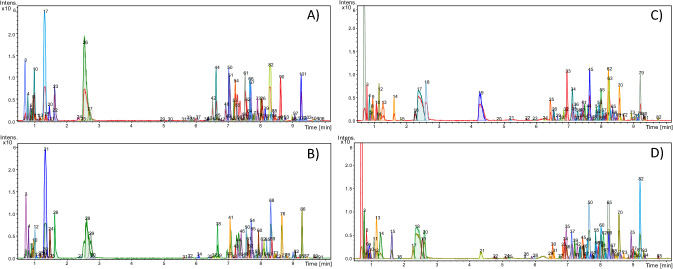
Total ion chromatogram (TIC) of representative samples after use of “dissect” algorithm. (**A**) Drought stress sample using UHPLC–ESI(−)–MS. (**B**) Control sample using UHPLC–ESI(−)–MS. (**C**) Drought stress sample using UHPLC–ESI(+)–MS. (**D**) Control sample using UHPLC−ESI(+)−MS.

In Fig. 1, a representative chromatogram of each group is shown. The data were acquired using UHPLC analysis and then treated with a “dissect” algorithm, where a list of compounds is created with averaged compound mass spectra making it possible to separate overlapping peaks. Based on the UHPLC gradient elution method, it is inferred that polar compounds are observed at 0–2 min, medium-polarity compounds at 2–6 min, and non-polar compounds at 6–10 min, all in the positive (UHPLC–ESI(+)–MS) and negative (UHPLC–ESI(−)–MS) ionization modes. A large number of chromatographic peaks after the dissect treatment was detected in both ionization modes, with an average peak count of 98 for UHPLC–ESI(−)–MS of drought samples, 96 for UHPLC–ESI(−)–MS control samples, 84 for UHPLC–ESI(+)–MS of drought samples, and 86 for UHPLC–ESI(+)–MS of control samples.

### Data analysis

In this study, a total of 32 chromatograms was acquired using UHPLC–MS, and then a manual comparison of spectra could easily lead to error. A series of chemometric methods were used to identify the metabolic differences among control and stressed plants. After data pre-processing, the statistic module of MetaboAnalyst was employed as the software for the analysis.

MetaboAnalyst 4.0 is a web-based tool suite for comprehensive metabolomics data analysis, interpretation, and multi-omics data integration^19,20^. MetaboAnalyst supports a wide array of functions for statistical, functional, as well as data visualization tasks. Some of the most widely used approaches include supervised classification techniques—PLS-DA—and unsupervised models—clustering analysis and heatmaps; besides the correlation between metabolites and metabolic pathways, all presented in this study.

#### Partial Least Square Discriminant Analysis (PLS-DA)

**Figure 2:**
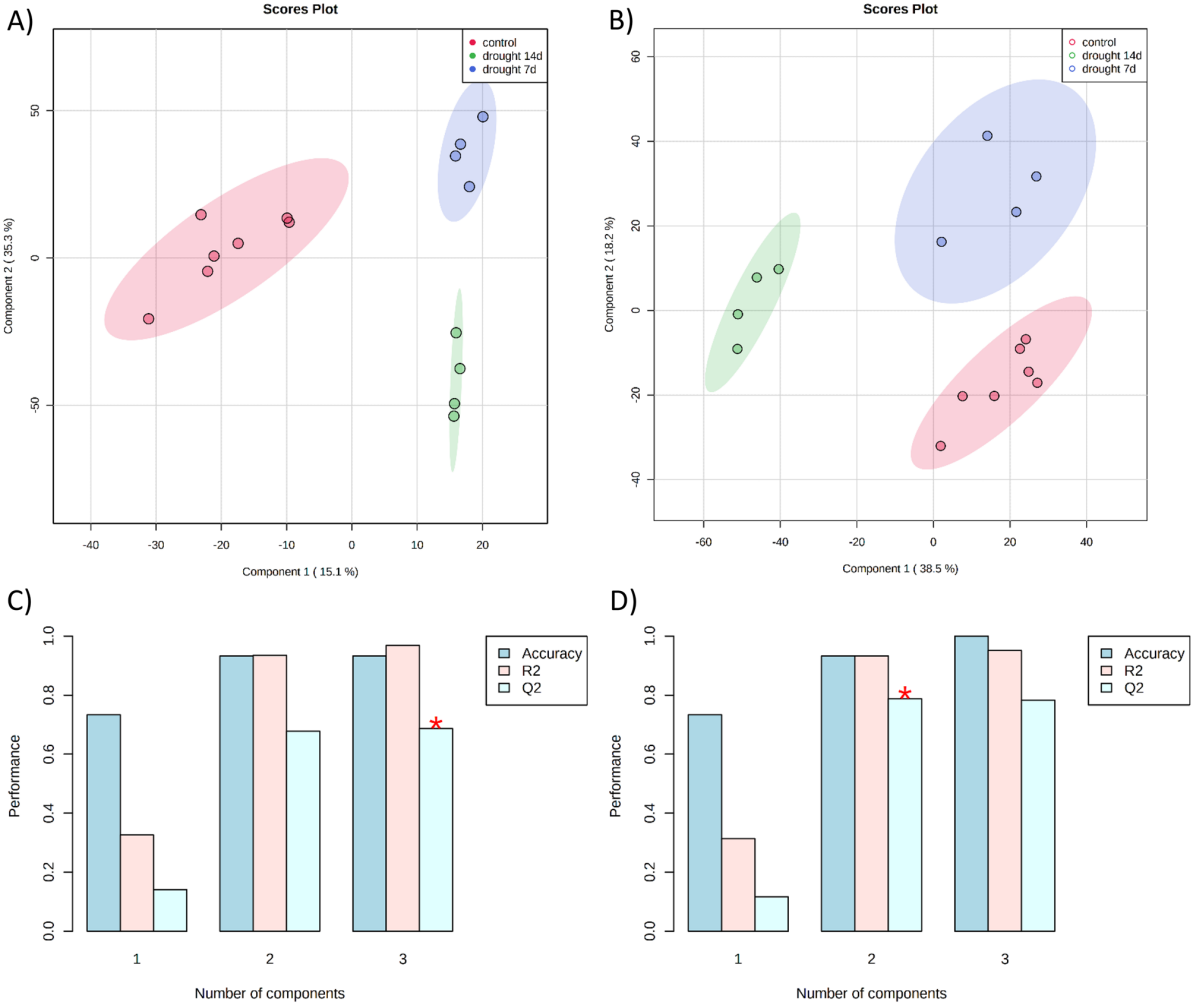
PLS-DA score plots comparing drought/control groups and “leave-one-out” cross validation (LOOCV). (**A**) Positive mode PLS-DA scores plot. (**B**) Negative mode PLS-DA scores plot. (**C**) LOOCV in positive mode. (**D**) LOOCV in negative mode.

To identify patterns and differentially expressed metabolites between the groups, the PLS-DA was applied as the multivariate separation method. This supervised method provides a robust regression technique based on labeled samples to optimize group separation by a component rotation^21^. PLS discriminant analysis was applied when comparing control, drought stress of 7 days, and drought stress of 14 days (Fig. 2).

Both ESI(+)–MS and ESI(−)–MS datasets presented clear segregation between groups, showing that the metabolism is affected by water deprivation. The 7-day group was closer to the control group when compared to the 14 days group, indicating that metabolism changed gradually through time. Cross-validation is essential to ensure the model's robustness due either to the classificatory nature and inherent overfitting of the PLS analysis^21^. We used the leave-one-out cross-validation (LOOCV), and the Q_2_ was evaluated on three components, resulting in the following values: Q_2_ = 0.6866 and accuracy = 0.933 for ESI(+)–MS and Q_2_ = 0.7830 and accuracy = 1.00 for ESI(−)–MS data, which represents a robust and reliable model. In a supervised classification model, R_2_ and Q_2_ are the accuracy parameters, where they range from 0 to 1 (higher means better accuracy) and R_2_ represents the raw predictive accuracy. The Q_2_ value is obtained when the PLS model is built on a training set against a test set, and usually a Q_2_ value higher than 0.65 is considered substantial for the model predictability. The PLS-DA is a fitting-method for identifying metabolites differentially expressed through the variable importance in projection (VIP) value. A variable with a VIP value higher than one is potentially important in the model construction. In ESI(−)–MS, we found 1126 variables with VIP > 1. In ESI(+)–MS we observed 1069 variables with VIP > 1, and from those, 182 variables with VIP > 2.

#### Hierarchical clustering heatmap

**Figure 3:**
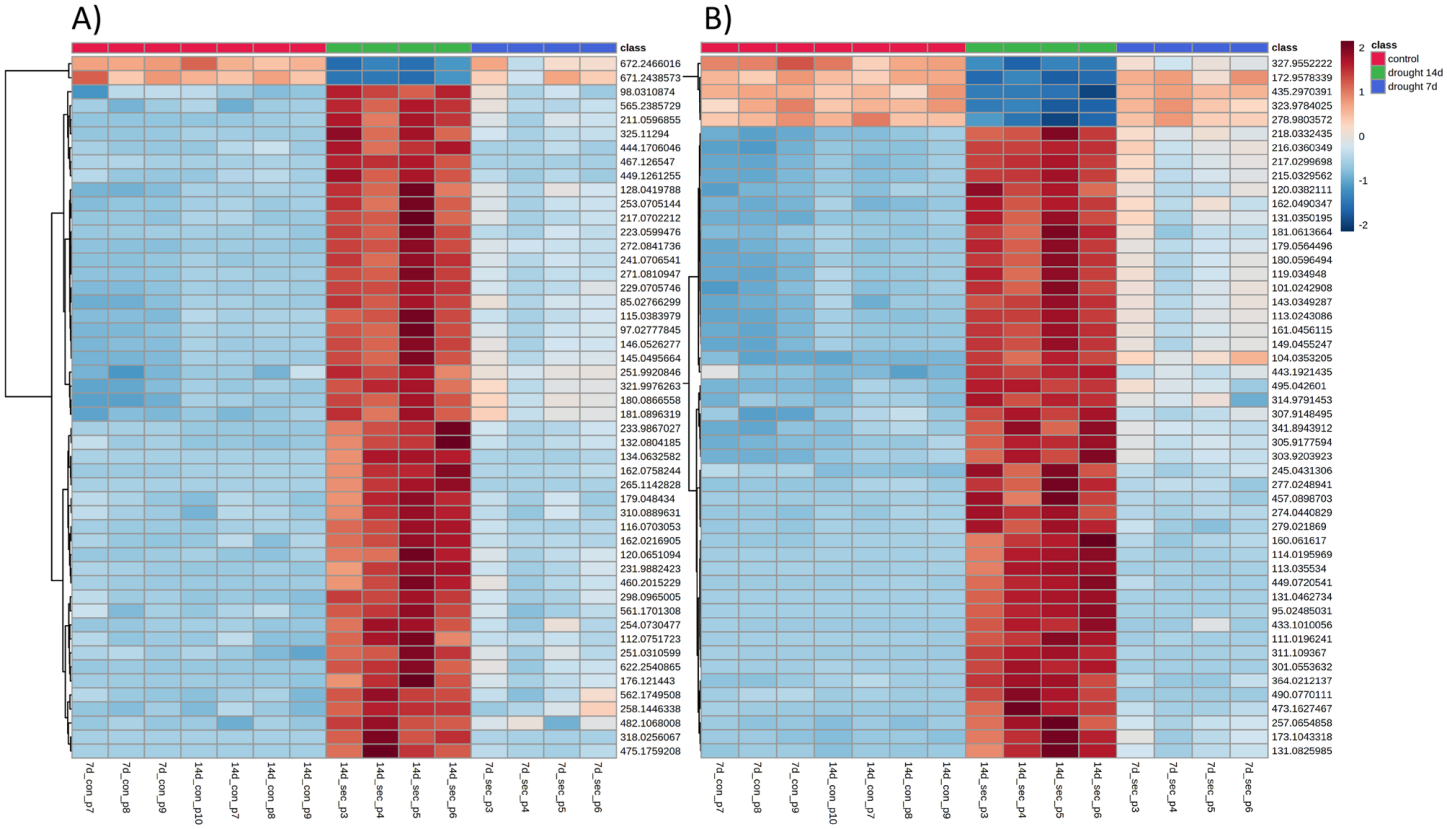
Heatmap analysis. Blue color indicates low intensity and red color indicates high intensity after the applied drought stress. The upper row represent sample groups, red: control group; green: 14 days of stress group; blue: 7 days of stress group. Top 50 VIP variables are shown on the right side. (**A**) UHPLC–ESI(+)–MS. (**B**) UHPLC–(−)–MS.

Figure 3 shows a heatmap generated using the top 50 variables showing the higher VIP values in each ionization mode analysis. The heat indicates the behavior of those variables throughout the samples.

It is possible to confirm the metabolic trends observed on PLS-DA using heatmaps as multivariate cluster analysis. A gradient is observed in metabolic intensity, increasing in most cases from the control group (the blue area in the left) up to the 14 days of drought stress (the red in the middle). For example, *m/z* 565.2385 has a low intensity on the control group, a medium intensity at 7 days of stress, and a high at 14 days of stress. This trend indicates a mass production of defense metabolites as a plant mechanism to survive and keep its metabolic functions in the presence of abiotic stress.

A few cases show an opposite trend, where metabolites went from a high intensity on control groups to a low one on the 14 days of the stressed group. For example, the detected ESI(−)–MS ions *m/z* 327.9555, 172.9578, 432.2970, 232.9784, and 278.9803 have high intensity on the control group, a medium intensity at 7 days of stress and a low intensity at 14 days of stress. This trend indicates that the drought stress can also cease metabolic production in those cases.

This heatmap cluster analysis shows that not only metabolite intensities can shift between groups with different treatments, those metabolites can be regulated according to the plants response to the stress applied.

#### Metabolic pathway correlation

This metabolomics study ends on the pathways most affected by drought stress. A clear and objective understanding of the affected-pathways is a way to get the information required to develop multiple biotechnological applications, where the development of stress-tolerant genotypes is the final goal to increase productivity. This type of study could also be part of a combined multiomics integration approach, together with genomics, transcriptomics and proteomics studies.

In recent metabolomics studies, many techniques have been applied in pathway correlation, from manual to automated methods^4,22–25^. Here, we used the mummichog algorithm^11,26^, based on over-representation analysis (ORA), to analyze UHPLC–MS data and predict enriched pathway activity, comparing the significant peaks of annotated metabolites.

**Figure 4:**
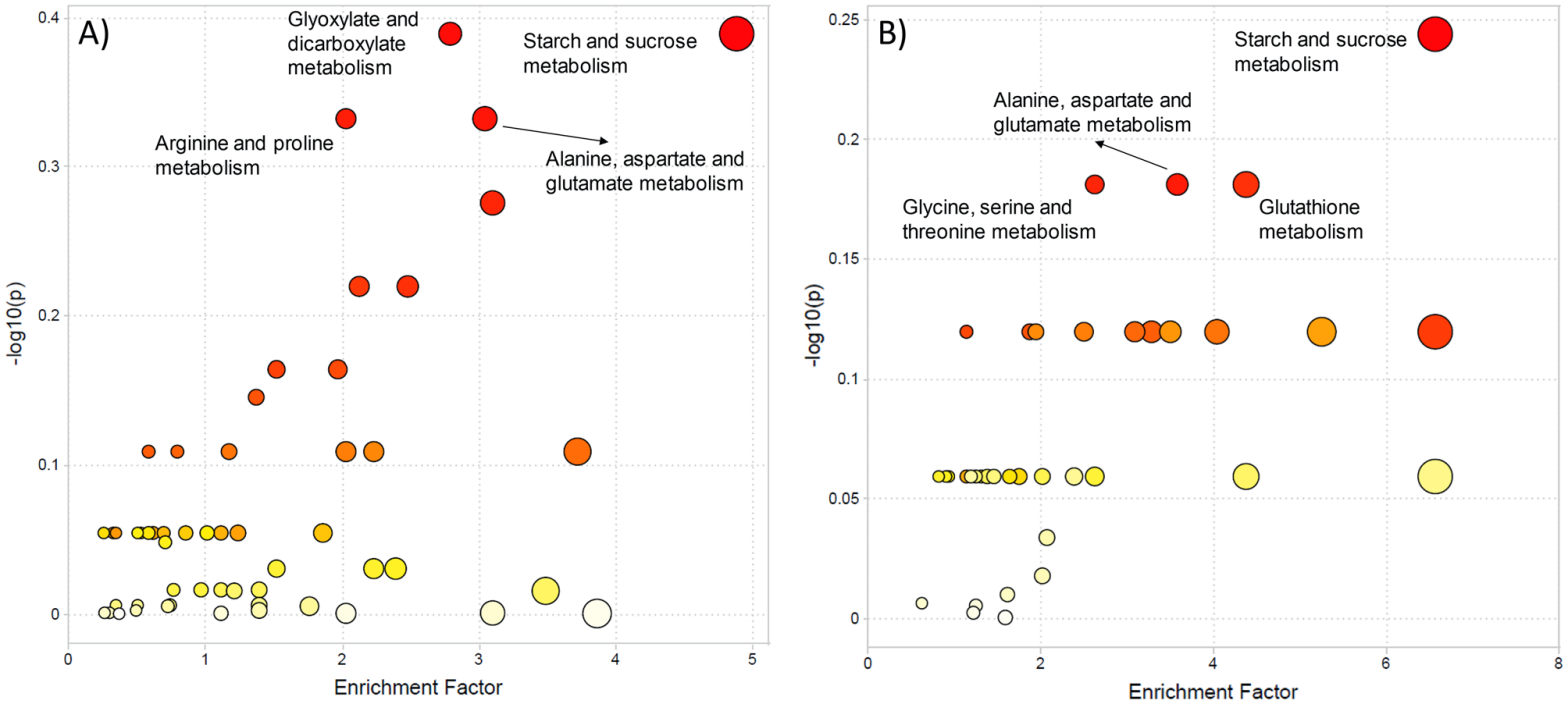
Metabolic pathway activity using the mummichog algorithm from (**A**) UHPLC–ESI(+)–MS and (**B**) UHPLC–ESI–(−)–MS data.

All samples from UHPLC–ESI(+)–MS and UHPLC–ESI(−)–MS were submitted to the “MS peaks to pathways” module of MetaboAnalyst. The pathway activity profile obtained is presented in Fig. 4, indicating the five most affected pathways in both ionization methods. In total, 176 and 85 metabolites from 42 pathways were significant upon applying the mummichog algorithm on UHPLC–ESI(+)–MS and UHPLC–ESI(−)–MS data, respectively. The “Supplementary material” (Tables S1 and S2) presents a list with all affected pathways.

In the UHPLC–ESI(+)–MS analysis, the most affected pathways were: starch and sucrose metabolism; glyoxylate and dicarboxylate metabolism; alanine, aspartate, and glutamate metabolism; and arginine and proline metabolism. And the most affected pathways in the UHPLC–ESI(−)–MS were: starch and sucrose metabolism; glutathione metabolism; alanine, aspartate, and glutamate metabolism; and glycine, serine, and threonine metabolism. Table 1 indicates the annotated metabolites that ensured the importance of the affected pathways.

**Table 1:** The most affected metabolic pathways in drought stress and metabolites associated.

Pathway	m/z	Retention time (min)	Adduct	Error (ppm)	KEGG compound	Compound name	Molecular formula
Starch and sucrose metabolism	343.1232	1.6	M + H	0.000240566	C00185	Cellobiose	C_12_H_22_O_11_
	219.0263	1.0	M + K	0.000148933	C00095 or C00031	d-Fructose or d-glucose	C_6_H_12_O_6_
	505.1755	1.6	M + H	0.000743043	C00721	Dextrin	(C_12_H_20_O_10_)_n_
	210.0337	7.7	M − 2H[2−]	0.000264633	C16688	Sucrose 6-phosphate	C_12_H_23_O_14_P
	503.1619	1.6	M − H	0.000140291	C00721	Dextrin	(C_12_H_20_O_10_)_n_
Glyoxylate and dicarboxylate metabolism	175.0232	2.6	M + H	0.000531391	C00417	*cis*-Aconitic acid	C_6_H_6_O_6_
	135.0284	1.4	M + H	0.000341869	C00149	Malic acid	C_4_H_6_O_5_
	148.0606	0.9	M + H	0.000150252	C00025	l-Glutamic acid	C_5_H_9_NO_4_
	129.0179	2.6	M − H_2_O + H	0.000333869	C00026	Oxoglutaric acid	C_5_H_6_O_5_
	119.0333	1.0	M + H	0.000546247	C00042	Succinic acid	C_4_H_6_O_4_
Alanine, aspartate and glutamate metabolism	129.0179	2.6	M − NH_3_ + H	0.000418284	C00940	2-Oxoglutaramic acid	C_5_H_7_NO_4_
	117.0177	0.7	M + H	0.000538683	C00122	Fumaric acid	C_4_H_4_O_4_
	146.0809	1.0	M − HCOOK + H	0.000166122	C03090	5-Phosphoribosylamine	C_5_H_12_NO_7_P
	325.0928	7.5	M + Cl[−]	0.001288772	C03406	l-Arginosuccinic acid	C_10_H_18_N_4_O_6_
	128.0352	0.9	M + ACN − H	8.38E-05	C00022	Pyruvate	C_3_H_4_O_3_
Arginine and proline metabolism	146.1648	0.6	M + H	0.00030209	C00315	Spermidine	C_7_H_19_N_3_
	148.0606	0.9	M + H	0.000150252	C05938	l-4-Hydroxyglutamate semialdehyde	C_5_H_9_NO_4_
	102.0544	0.9	M − CO + H	0.000409762	C04281	l-1-Pyrroline-3-hydroxy-5-carboxylate	C_5_H_7_NO_3_
	146.0809	1.0	M + H	0.00022109	C02946	4-Acetamidobutanoate	C_6_H_11_NO_3_
	119.0333	1.0	M − CO_2_ + H	0.000575491	C05946	d-4-Hydroxy-2-oxoglutarate	C_5_H_6_O_6_
Glutathione metabolism	657.1498	8.3	M + HCOO	0.000240802	C00127	Glutathione disulfide	C_20_H_32_N_6_O_12_S_2_
	128.0352	0.9	M − H_2_O − H	5.26E − 05	C00025	l-Glutamic acid	C_5_H_9_NO_4_
Glycine, serine and threonine metabolism	146.0461	0.9	M + ACN-H	0.000182687	C00258	Glyceric acid	C_3_H_6_O_4_
	233.9926	0.9	M + Cl	0.000842469	C01102	O-Phospho-l-homoserine	C_4_H_10_NO_6_P

The starch and sucrose metabolism was the most affected pathway in either analyses, ESI(+)–MS and ESI(−)–MS. This metabolic pathway has a role in photosynthesis, when sucrose and starch are converted from triose-phosphates during the CO_2_ plant fixation, with strict governance between both processes. Synthesis of sucrose and starch occurs, respectively, at the cytosol and chloroplast, and the Pi-triose phosphate antiport system mediates the coordination^27^. Triosephosphate synthesis is affected by a slow sucrose production that results in low Pi available to the chloroplast, while a rapid sucrose production results in the removal of triose phosphate in excess. Morphologically, plants with deficient sucrose synthesis present reduced growth and tolerance to anaerobic-stress conditions^28^.

Glyoxylate and dicarboxylate metabolism is an important abiotic stress-related pathway, providing a balance in metabolic disorders to improve tolerance^29^. The glutamic acid, indicated in Table 1 and present in both glyoxylate and glutathione metabolism, is vastly transported in phloem sap and plays a major role in many biosynthesis of other amino acids, chlorophylls, and tricarboxylic acid. The glutamate synthase (GS) isoforms GS1 and GS2 are described as pivotal enzymes used in genetically enhanced species to improve photorespiration capabilities^30^ and response to energy supply^31^.

The alanine, aspartate, and glutamate metabolism is considered a short catabolic pathway, where an alanine is converted into pyruvate, which was highly affected in our study. There are essential metabolic branches influenced by this pathway in mitochondrial multi-enzyme system, such as isoleucine, cysteine, methionine, and threonine synthesis, which clearly states its importance from a nutritional perspective^32^.

The arginine and proline pathway is related to nitrogen metabolism in plants, essential for production of nucleic acids and proteins. Arginine is a precursor of polyamines and has a role in proline biosynthesis when glutamate is not available. The influence of drought stress is highly expected in this pathway, given that proline has the capability of protein protection and membrane structure in dehydration cases^33^, acting on redox status or as a scavenger of reactive oxygen species that could increase cellular solute concentration.

Many studies on metabolites from glycine, serine, and threonine metabolism, looking for a better understanding of the chemical defenses against salt, cold, and drought stresses in plants, are available. For instance, some of them show that threonine metabolites are involved in plant growth and development, cell division, and phytohormones regulation^34,35^.

## Materials and methods

### Chemicals

Methanol UHPLC grade, acetonitrile LC–MS grade, methyl-tert-butyl-ether, formic acid LC–MS grade, and sodium hydroxide ACS grade were purchased from Sigma-Aldrich (Merck, USA). Water was obtained using a Milli-Q system (Millipore, USA).

### Plant material and growth conditions

The oil palm plants used were clones regenerated out of embryogenic calluses obtained from leaves of an adult plant belonging to the *E. guineensis* genotype AM33^12^. The AM33 genotype is a plant from a commercial field in the State of Pará, in Brazil. This field was established with seeds from a cultivar developed by Embrapa. Oil palm seeds produced and commercialized by Embrapa in Brazil are “Deli x La Mé”, and the parentals came from progenies obtained from Dura and Tenera plants self-crossed. Plants were kept in black plastic pots (5 L), containing 1700*g* of a mix of vermiculite, soil, and a commercial substrate (Bioplant, Brazil) in a 1:1:1 ratio—on a dry basis—and fertilized using 2.5 g/L of the formula 20–20–20.

Before starting the experiments, we screened the plants to standardize the developmental stage, size, and the number of leaves. The experiment was performed in a greenhouse at Embrapa Agroenergy (www.embrapa.br/en/agroenergia), in Brasília, DF, Brazil (S-15.732°, W-47.900°). The plant material collection and methodology used in this study complied with relevant institutional, national and international guidelines and legislation. The main environmental variables (temperature, humidity, and radiation) fluctuated according to the weather conditions and were monitored throughout the experimental period from the data collected at a nearby weather station (S-15.789°, W-47.925°).

### Experimental design and drought stress

The experiment consisted of two treatments—control and drought-stressed plants—with four plants kept in a substrate in the field capacity (control), and six plants submitted to drought stress. The young oil palm plants were subjected to treatments when they were in the growth stage known as “bifid” saplings. Drought stress consisted of total suppression of irrigation for 14 consecutive days. At the end of this period, the substrate water potential, as measured by the water potential meter Decagon mod. WP4C (Decagon Devices, Pullman, WA, USA), was 0.19 ± 0.03 MPa (control) and − 13.61 ± 1.79 MPa (drought stress), while the relative water content of leaves was 90.50 ± 0.95% (control) and 49.18 ± 9.76% (stressed plants). Before the onset of drought stress, oil palm leaves had the highest gas exchange rates, as measured by infrared gas analyzer Li-Cor model 6400XT (Li-Cor, Lincoln, NE, USA). Under drought, leaf gas exchange rates in drought-stressed plants dropped to negligible values (data not shown).

Leaf samples were collected at 7 and 14 days after the onset of the stress from four control plants and four stressed plants. Leaf samples with approximately 50 mg were collected for the metabolomics analysis; four replicates per plant. After harvesting, samples were immediately frozen in liquid nitrogen and stored at − 80 °C until metabolites extraction and analysis.

### Metabolites extraction

Each sample was ground in a ball mill (Biospec Products, USA) before solvent extraction. Metabolites were extracted using an adapted protocol from The Max Planck Institute^36^, called "All-in-One", which provides a polar fraction for secondary metabolite analysis, a nonpolar fraction for lipidomics and a protein pellet for proteomics; all obtained from the same plant sample. Each ground sample was added to a microtube and mixed with 1 mL of a methanol and methyl-tert-butyl-ether (1:3) solution at − 20 °C. After homogenization, they were incubated at 4 °C for 10 min. Each microtube was ultrasonicated in an ice bath for another 10 min. Then, 500 μL of a methanol and water (1:3) solution was added to the microtube before centrifugation (12,000 rpm at 4 °C for 5 min). Three phases were separate: an upper non-polar (green), a lower polar (brown), and a remaining protein pellet. Samples were transferred to fresh microtubes and vacuum-dried in a speed vac (Centrivap, Labconco, Kansas City, MO, USA) overnight at room temperature (~ 22 °C).

### UHPLC–MS

A total of 0.4 μL of the extract was then resuspended in 850 μL of methanol and water (1:3) solvent mixture and then analyzed by UHPLC–MS. The Nexera X2 UHPLC system (Shimadzu Corporation, Japan) was equipped with a reversed-phase Acquity UPLC BEH C8 column (1.7 μm, 2.1 × 150 mm) (Waters Technologies, USA). Chromatographic run parameters were: isocratic from 0 to 0.5 min (4% B), linear gradient from 0.5 to 10 min (34% B) and 10–15 min (100% B) and isocratic from 15 to 18 min (100% B). Solvent A was 0.1% formic acid in water (v/v), and solvent B was 0.1% formic acid in acetonitrile (v/v). The flow rate was set at 400 μL/min.

High-resolution mass spectrometry (HRMS) was performed in a MaXis 4G Q-TOF MS system (Bruker Daltonics, Germany) using an electrospray source in the positive and negative ion modes (ESI(+)–MS and ESI(−)–MS). The MS instrument settings used were: endplate offset, 500 V; capillary voltage, 3800 V; nebulizer pressure, 4 bar; dry gas flow, 9 L/min, dry temperature, 200 °C; and column temperature, 40 °C. The acquisition spectra rate was 3.00 Hz, monitoring a mass range from 70 to 1200 m/z. Sodium formate solution (10 mM NaOH solution in 50/50 v/v isopropanol/water containing 0.2% formic acid) was directly injected through a 6-port valve at the beginning of each chromatographic run to external calibration. UHPLC–MS data was acquired by the HyStar Application version 3.2 (Bruker Daltonics, Germany), and data processing was done using Data Analysis 4.2 (Bruker Daltonics, Germany). This extraction method and UHPLC–MS analysis system has been optimized and used in recent studies from our group^4^ and resulted in reliable results, therefore is replicated in the present work.

### Data analysis

The raw data from UHPLC–MS was exported as mzMXL files using DataAnalysis 4.2 software (Bruker Daltonics, Germany) and pre-processed using XCMS Online^37,38^ for feature detection, retention time correction, and alignment of metabolites detected on UHPLC–MS analysis. Two datasets, one for the samples harvested at 7 days of drought and another for the samples harvested at 14 days, were created.

Pre-processing done using optimized parameters based on Albóniga et al.^39^, which tunes feature detection to obtain a smaller data matrix but with a higher number of variables with an SD < 20%, which creates a more robust data processing. Peak detection was performed using centWave peak detection (Δ m/z = 25 ppm; mzdiff = 0.002; minimum peak width = 12 s; maximum peak width = 40 s) and mzwid = 0.02, minfrac = 0.16, bw = 1 were used for retention time alignment. Statistics analysis used an unpaired parametric t-test (Welch t-test).

The processed data (csv file) was then submitted for analysis in the MetaboAnalyst 4.0^19,20^. Before multivariate analysis [partial least square discriminant analysis (PLS-DA), heatmap, and hierarchical cluster analysis (HCA)], all data variables were normalized by internal standard (sodium formate adduct, rT = 0.1 min; *m/z* 226.9522 in positive mode, *m/z* 316.9478 in negative) and scaled by the auto-scaling method. A PLS model was built with three groups to attempt the segregation between control (irrigated) and stressed samples (7 days and 14 days of drought). Internal validation—leave-one-out cross-validation (LOOCV)—was performed to ensure model robustness. The results described here were obtained at the MetaboAnalyst web tool in 4/14/2020.

A heatmap was built using all samples and the following criteria: distance measure, Euclidean; clustering algorithm, Ward; standardization, autoscale; and top 25 features using t-test/ANOVA to retain the most contrasting patterns.

The last step of the data processing was the use of the mummichog algorithm approach^11^ in the MS peaks to pathways module of MetaboAnalyst. The criteria used on this analysis were: molecular weight tolerance, 5 ppm; primary ions enforced; p-value cutoff, 0.01; pathway library, *Oryza sativa japonica* (Japanese rice) from Kyoto Encyclopedia of Genes and Genomes (KEGG)^40–42^.

## Conclusion

Through an untargeted metabolomics method, different peak intensities between control and stressed groups were used as the main parameter to evaluate tolerance levels to water deficit and to screen drought tolerance in *E. guineensis* leaves.

A high amount of metabolites and pathways were significantly affected by drought stress. We detected metabolites from a wide range of chemical classes using UHPLC–MS as a high-throughput untargeted method and putatively annotated 24 differentially expressed metabolites from the most affected pathways on ESI(+)–MS and ESI(−)–MS. These pathways were: starch and sucrose metabolism; glyoxylate and dicarboxylate metabolism; alanine, aspartate, and glutamate metabolism; arginine and proline metabolism; and glycine, serine, and threonine metabolism.

Metabolic pathways and their respective compounds, presented in this study, corroborated with the clear metabolic response of *E. guineensis*, given that most of those pathways are known by their importance in response to abiotic stress, such as drought stress. These results implicate a more accurate and responsive multi-omics future study targeting enhanced crops with a higher tolerance to water deficit, resulting in an improved crop yield.

## Supplementary Information

Supplementary Tables.
